# Extracorporeal Shockwave Therapy for Chronic Venous Ulcers: A Randomized Controlled Trial

**DOI:** 10.31661/gmj.v10i0.1931

**Published:** 2021-04-25

**Authors:** Parisa Taheri, Morteza Shahbandari, Mehrnoosh Parvaresh, Babak Vahdatpour

**Affiliations:** ^1^Department of Physical Medicine and Rehabilitation, School of Medicine, lsfahan University of Medical Sciences, lsfahan, Iran; ^2^Department of Vascular Surgery, Isfahan University of Medical Sciences, lsfahan, Iran

**Keywords:** Chronic Venous Ulcer, Extracorporeal Shockwave Therapy, Compression Bandaging

## Abstract

**Background:**

Chronic venous ulcers (CVUs), demanding specialized care, are still a major socioeconomic problem facing health care systems worldwide. This study’s main goal was evaluating the efficacy of ESWT application as an AT in the treatment of wounds for curing CVUs.

**Materials and Methods:**

50 patients presenting with CVUs were divided into two groups of ESWT and control randomly. Then, ESWT was applied one session per week, during four weeks, along with routine CB. The control group also received sham ESWT together with CB. In this respect, pain score, wound size, patient satisfaction and quality of life (QoL) using the Charing Cross Venous Ulcer Questionnaire (CCVUQ) were consequently assessed at baseline, week four, and week eight and then compared between both groups.

**Results:**

The findings showed that patients receiving ESWT along with CB had significantly lower pain and were also feeling more satisfied than the cases undergoing CB alone (P<0.05). The significant efficiency of ESWT in improving the healing process of CVUs was further observed (P<0.05). In addition, QoL, assessed by the CCVUQ, was significantly higher in patients receiving ESWT (P<0.05).

**Conclusion:**

These findings established that ESWT was a feasible and safe option to treat patients presenting with CVUs in another word,ESWT seems to be a safe and effective adjunct therapy (AT) compared with CB in patients with CVUs.

## Introduction

Chronic venous ulcers (CVUs) are open complex wounds caused by chronic venous insufficiency (CVI) in the lower extremity below the knee on the leg or the foot that have been present for at least six weeks [1]. The frequency of these wounds is rising, associated with impaired quality of life (QoL), reduced mobility, pain, stress, and loss of dignity. herefore, such ulcers demand specialized care that may challenge patients and medical teams, as a major socioeconomic problem facing health care systems worldwide [2,3]. The management of ulceration is accordingly dependent on the associated causes, and it can include both conservative and surgical options. Multi-layer compression bandaging (MLCB), hyperbaric oxygen therapy (HBOT), CVI surgery, radiofrequency (RF) radiation, endovenous laser treatment (EVLT), and sclerotherapy (ST) are thus among well-known minimally invasive procedures for the management of these ulcers [4]. The MLCB, that aims to improve venous returns and to reduce venous hypertension, has been so far acknowledged as an effective standard treatment in the management of CVUs [5,6]. The healing rates for such wounds within six months of MLCB in some specialist clinics has been also reported to be around 70% [6], whereas, other studies have reported the rate of unhealed CVUs up to 50% after two years [7]. Therefore, alternative therapies may be desirable, because of long treatment time and rate of unhealed wounds, since they accelerate the healing of refractory wounds with multimodal treatments, improve patients’ health-related QoL, and minimize health care expenses [8]. In this regard, extracorporeal shockwaves are low-energy pulse waves that have been clinically implemented as an effective treatment of urinary stones as well as some orthopedic and traumatic indications over the past decades [9,10,11,12,13]. Recently, extracorporeal shock wave therapy (ESWT) has been exercised to treat acute and chronic non-healing wounds [8,13] and it is believed that this modality induces neovascularization and mechanical stimuli causing proliferation of a number of cells including osteoblasts [14]. The effectiveness of ESWT on treatment of chronic wounds with different etiologies has been further reported in previous studies. Hence, chronic wounds such as CVUs in DM patients, pressure ulcers, and PAD have demonstrated promising healing signs [15,16,17,18,19]. Having no control group, selecting patients with different causes of chronic wounds, and recruiting a relatively low number of samples have been among limitations of prior studies. In this respect, Zhang et al., had demonstrated that the healing process of chronic lesions, compared with the standard care treatment alone, could be significantly augmented by adding ESWT as an adjunct therapy (AT). Among the limitations of this systematic review was that the subjects involved in the selected studies were patients with chronic wounds irrespective of their etiologies [20]. Therefore, this research designed to compare the efficiency of ESWT along with CB versus CB alone in the healing of CVUs.

## Materials and Methods

The current double-blind parallel-group randomized clinical trial in a multi-centered design was conducted on the patients with chronic venous ulcers in hospitals of Isfahan, Iran, in 2018-2019. A venous ulcer was characterized as a split in the epithelial surface in the gaiter region, with proof of reflux of greater than 0.5 seconds in the venous system. Besides, an over two-week ulcer span with a size of more than 1 cm2 was required for trial purposes [21]. The sample size included 50 patients (25 in the intervention group receiving ESWT along with routine treatment and 25 in the control group receiving sham ESWT along with routine treatment), who met the study inclusion criteria and selected by random sampling method. The inclusion criteria were over 18 years of age, the presence of leg ulcer due to underlying venous insufficiency, the ulcer size of greater than 1 cm2 and the persistence of the ulcer for more than 6 weeks. The exclusion criteria were the history of vascular surgery in the last 6 months, the presence of rheumatic arthritis or systemic vasculitis, acute deep vein thrombosis, coagulopathy, tumors, diabetes, cardiovascular disease, kidney disease, ESWT contraindications such as severe arterial hypertension, coagulopathy and anticoagulant therapy, pregnancy, ESWT intolerance, and contraindications to ESWT (severe arterial hypertension; anticoagulant therapy; wound infection during treatment and the need for antibiotics or a change in treatment regimen).

Prior to implementation, this study was approved by the Research Council of MUI (Ethic Number: IR.MUI.MED.REC.1397.060), and then registered on the IRCT (IRCT20190908044730N2). This research study clarified its objectives and the main principles to the entire participants to ensure their data confidentiality and fulfill the ethical commitments. Also, the study collected informed consent forms from all patients. Besides, participants had the freedom to terminate their participation in any stage of the study. After obtaining informed consent, 50 patients with chronic venous ulcers were divided into two groups of 25, the intervention group (receiving ESWT: MP 100, Storz Medical, Switzerland) and the control group (receiving sham ESWT) using random allocation software version 2 and block randomization method. In the intervention group, the extracorporeal shock wave therapy was performed in addition to routine compression bandaging once a week for 4 weeks. Each ESWT session consisted of 100 pulses per square centimeter of wound area. The total energy for each pulse was 3.5 mJ and the frequency was 5 Hz. The control group did not receive the waves (the device was turned off for this group for blinding and used as a sham), and only routine compression bandaging was performed for patients. The electromagnetic device (DUOLITH SD1 standard device, Storz Medical, Tägerwilen, Switzerland) was used to perform the shock. The method of blinding in this study was that participants were treated with the device with a four-layer dressing. But for the control group, in addition to the four-layer dressing, the off device was used. The analyzer was also unaware of the blinding conditions.

In order to collect data and evaluate the ulcers at the baseline, the patients were visited at 4 and 8 weeks. The background characteristics of patients included age, gender, BMI and wound duration. The main outcomes included patients’ quality of life, wound size (cm2), amount of drainage (no, low, moderate, high), patient satisfaction and pain intensity. Patients’ quality of life was assessed using the English version of Charing Cross Venous Ulcer Questionnaire (CCVUQ). The validity and reliability of this questionnaire were evaluated by Smith et al. in 2000 and showed acceptable reliability using internal consistency (Cronbach’s alpha=0.93) and test-retest analysis (r=0.84). The CCVUQ is composed of 20 items distributed in four subscales of social interaction, domestic activities, emotional state, and aesthetics. The questions are set on a Likert scale from 1 to 5 and the total scores of the questionnaire ranged from 20 to 100. A low CCVUQ score on the whole scale or any subscale indicates a better quality of life [21]. This questionnaire was completed by the researcher. Visual Analogue Scale (VAS) was used to assess pain intensity. The VAS is a simulated self-report visual scale. In this study, the VAS was a simple ruler numbered from zero (no pain) to 10 (maximum pain intensity). After explaining about this tool, the patients were asked to determine the intensity of their pain according to the given explanations [22]. The collected data were analyzed by SPSS version 24 software (SPSS Inc. Chicago, Illinois, USA) using descriptive statistics, Fisher’s exact test to compare qualitative variables between the two groups, repeated measures ANOVA to compare of the two groups at different times, and independent t-test to compare the means between the two groups at a statistically significance level of P-value<0.05 for all tests.

## Results

**Figure 1:**
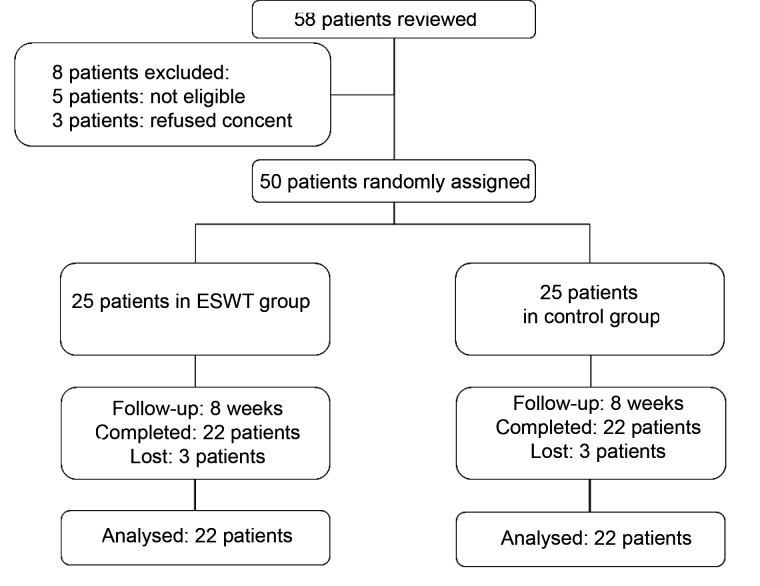
CONSORT flowchart

 According to (Figure-1), 58 patients were examined in this study, three patients had no informed consent to participate in this project, five patients were not eligible to participate in the study after initial studies, and three patients in the ESWT group and three patients in the control group were excluded from the study during follow-up and were reluctant to continue cooperation. The final analysis was performed on 22 patients in the ESWT group and 22 patients in the control group. Of the 22 participants in the ESWT group, 20 were male (91%) and 2 were female (9%). Out of 22 patients in the control group, 19 were male (86%) and 3 were female (14%). There was no significant difference in the gender distribution between the two groups (P=0.635). The mean age was 56.1±15.1 years in the ESWT group and 57.3±11.8 years in the control group. There was also no significant difference in age between the two groups (P=0.756). Other background information of the patients is reported in Table-1. Based on the results of Table-1, no significant difference in contextual variables was observed between the two groups (P>0.05). In this study, the amount of wound drainage was investigated observationally and qualitatively, the results of which are reported in Table-2. Based on this analysis, no significant difference in the amount of drainage in any of the time periods was observed between the two groups (P>0.05). 

**Figure 2:**
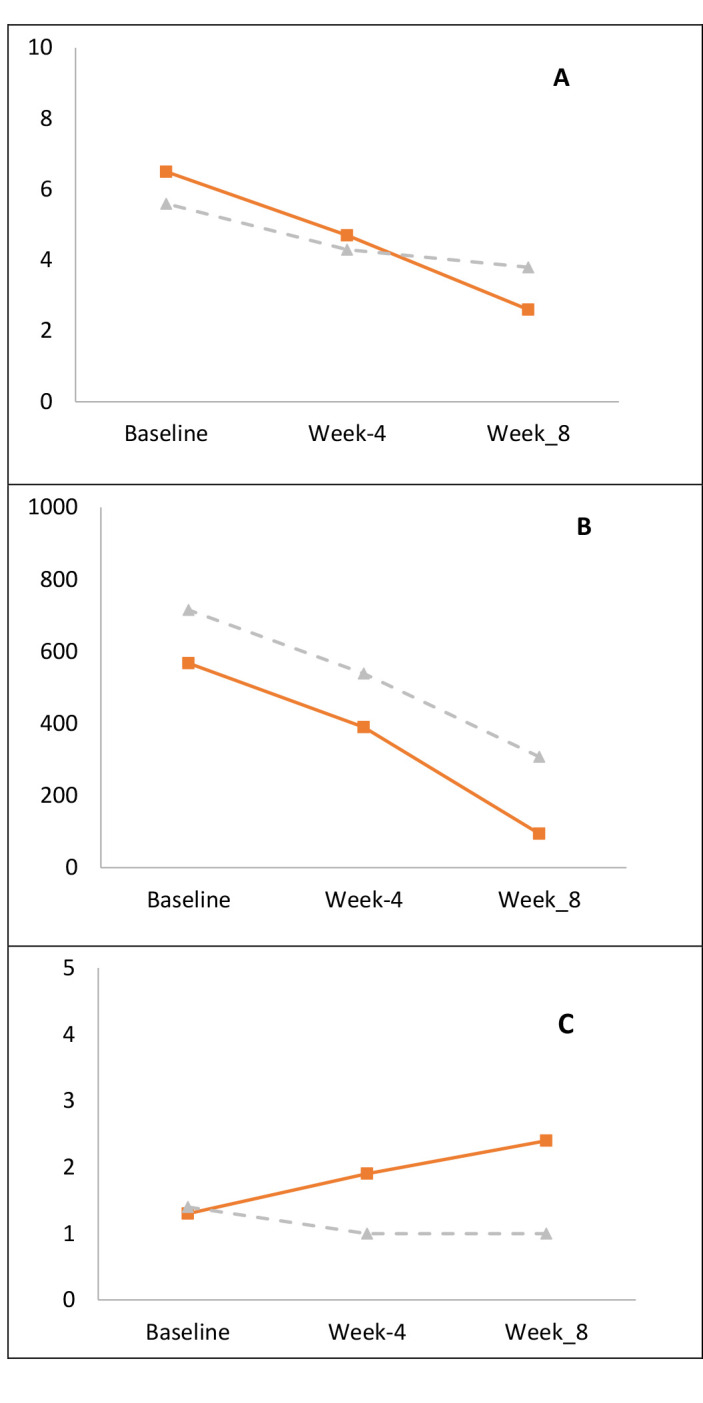
Trend of studied outcomes during study period between groups (A: Pain score, B: Wound size, C: Satisfaction score)

**Figure 3:**
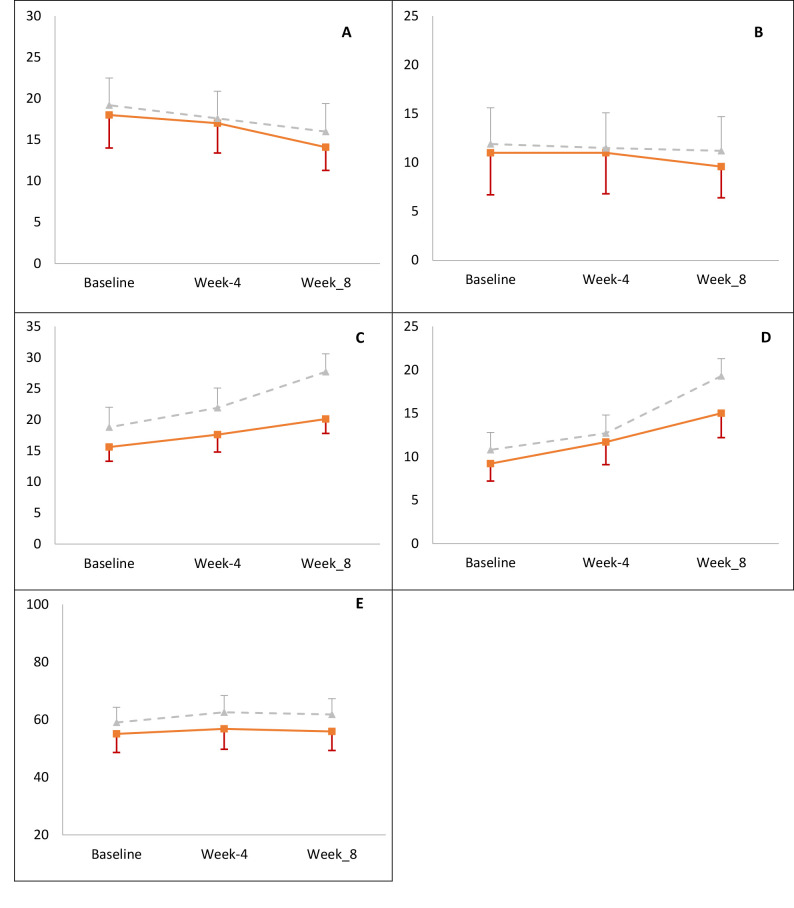
Trend of CCVUQ score during study period between groups (A: Social interaction domain score, B: Domestic activities domain score, C: Aesthetics domain score, D: Emotional state domain score, E: CCVUQ total score)

In this study, ulcer size, patient satisfaction and pain intensity were evaluated by repeated measures ANOVA test, the results of which are reported in Table-2 and (Figure-2). Based on the results, only the patient satisfaction index exhibited a significant difference between the two groups, so that the ESWT group had more satisfaction than the control group (P<0.001), but pain intensity and ulcer size in the two groups showed no significant difference (P>0.05). In this study, the patient’s quality of life in general and separately into four subscales (social interaction, domestic activities, emotional state, and aesthetics) was evaluated by the repeated measures ANOVA test between the two groups, the results of which are reported in (Table-2) and (Figure-3). The results of the analysis indicated the two groups represented a considerable difference regarding three subscales, including aesthetics, emotional state as well as the total CCVUQ, whose values, in comparison with the control group, were elevated in the ESWT group; however, subscales differed no significantly concerning the social interaction and domestic activities (P>0.05).

## Discussion

Given the significant effects of chronic wounds on patients’ comfort and QoL, any interventions accelerating their healing process will be considerable. ESWT, as a new therapeutic option, has been recently exercised in the treatment and control of such ulcers [15,20]. This study’s main goal was evaluating the efficacy of ESWT application as an AT in the treatment of wounds for curing CVUs. The findings showed that patients receiving ESWT along with CB had significantly lower pain and were also feeling more satisfied than the cases undergoing CB alone. The significant efficacy of ESWT in improving the healing process of CVUs was further observed. In addition, QoL, assessed by the CCVUQ, was significantly higher in patients receiving ESWT. These findings established that ESWT was a feasible and safe option to treat patients presenting with CVUs. The existing evidence indicates that the use of ESWT can be safe and effective in the control and treatment of chronic wounds. In this respect, Wolff et al. had conducted an RCT to evaluate the impact of ESWT in 282 patients with unsuccessfully treated chronic wounds and had found that positive treatment had been achieved in 74.03% of the cases [15]. In a clinical study, Saggini et al. had similarly assessed the use of ESWT along with regular conservative dressings to treat 40 patients affected with chronic post-traumatic, venous, and diabetic foot ulcers, had reported significant improvements in wound size, pain score, and healing process in patients undergoing ESWT compared with the controls [16]. In one other study by Schaden et al., a total number of 208 patients with a variety of acute and chronic wounds (including traumas, post-operative wounds, venous or arterial insufficiency, pressure necrosis, or burns) had been treated through a combination of debridement, ESWT, and moist wound dressings. Reports indicated a 75% treatment completion rate among patients, representing the feasibility of ESWT. Also, it was concluded that ESWT was tolerable by patients having soft tissue wounds with acute and chronic states [17]. In this line, 28 patients with CVUs had been assessed in a pilot study by Cooper to investigate the use of ESWT combined with MLCB as a standard practice. The level of QoL, wound healing rate, and pain scores in the patients had significantly improved after six sessions of treatment with two-week intervals compared with the baseline [19]. A recent review studying ESWT effectiveness as an AT for the chronic wound treatment had used seven CRTs involving 301 subjects. The results of meta-analyses indicated that the application of ESWT could considerably speed up the weakened, inefficient healing process of such wounds, despite the suggestions to conduct more high-quality trials to assess ESWT efficacy in chronic wound treatment since the reviewed research were limited and had a small sample size [20]. The results of this study represent the considerably positive, reducing impacts of using ESWT as a routine AT for wound treatment on pain, wound size, QoL, and patient satisfaction with CVUs. Due to some potential limitations and the wide variety of ESWT protocols practiced in different studies and even if the related literature has thus far confirmed the positive effect of ESWT for such wounds; further investigations, based on various protocols and follow-ups are necessary to clarify the healing impacts of ESWT on chronic wounds for detecting the optimal procedures depending on the type of wound and etiology. The mechanism of ESWT in the treatment of chronic wounds has remained unclear; however, some mechanisms are suggested to improve wound healing. Many studies using animal models have thus far reported more significant levels of HIF-1-alpha and VEGF and hypoxia-inducible factor 1-alpha, which exist in the normal trend of post-treatment wound healing [23,24,25]. On the other hand, post-ESWT increased angiogenesis and cell activity can lead to improvements in tissue regeneration and accelerate wound healing process [26]. In addition, it has been established that ESWT boost extracellular signal-regulated kinase 1/2 (ERK1/2) by activating purinergic receptors in response to the release of cellular ATP [27]. Although its mechanism is not widely understood but some advantages of ESWT such as non-invasiveness, no serious complications, superiority to other standard treatments, and cost-effectiveness [12] can make this modality considerable as an easy method to implement for clinicians and acceptable for the majority of patients with positive wound healing outcomes. One of the limitations of this study is the ESWT-induced pain, which initially caused patients to suffer, but after obtaining the results of the intervention, they expressed their satisfaction. Moreover, due to the heterogeneity of ulcer shape in the patients, there was a possibility of error in measuring the ulcer size.

## Conclusion

 The finding of this research demonstrated that ESWT as an AT for chronic wound treatment could be effective in treatment and control of CVUs and result in improvements in wound healing, higher levels of satisfaction in patients, pain reduction, and a better QoL. More CRTs are thus needed to assess the exact efficacy of ESWT in these patients.

## Acknowledgment

 We are sincerely thankful to Isfahan University of Medical Sciences.

## Conflict of Interest

 None.

**Table 1:** Baseline Patient Characteristics (mean ± SD)

** Variables**	ESWT* group (n=22)	**Control group (n=22) **	** P-value**
**Weight (kg)**	86.0 ± 14.9	89.4 ± 14.2	0.445
**High (cm)**	172.0 ± 6.7	174.3 ± 5.0	0.199
** Body Mass Index**	29.0 ± 4.0	29.4 ± 4.4	0.756
** Wound duration (week)**	62.0 ± 56.0	57.3 ± 3.6	0.741

*ESWT: Extracorporeal Shock Wave Therapy.

**Table 2:** Comparison of Pain Score, Wound Size, Satisfaction Score and CCVUQ Findings between Groups.

**Variables **	**ESWT group (n=22)**			**Control group (n=22)**			**P-value**
	**Base line**	** Week 4**	** Week 8**	** Base line**	** Week 4**	**Week 8**	
** VAS pain score**	=6.5 ± 2.5	4.7 ± 1.9	2.6 ± 1.4	5.6 ± 1.8	4.3 ± 1.3	3.8 ± 1.6	0.860
** Wound size**	567.8 ± 644.2	390.2 ± 467.3	94.8 ± 176.2	715.8 ± 769. 7	539.4 ± 627.3	307.6 ± 433.6	0.281
** Satisfaction score**	1.3 ± 0.63	1.9 ± 0.57	2.4 ± 0.66	1.4 ± 0.96	1.0 ± 0.62	1.0 ± 0.57	<0.001
** CCVUQ findings**
** Social interaction**	18.0 ± 4.0	17.0 ± 3.6	14.1 ± 2.8	19.2 ± 3.3	17.6 ± 3.3	16.0 ± 3.4	0.191
** Domestic activities**	11.0 ± 4.3	11.0 ± 4.2	9.6 ± 3.2	11.9 ± 3.7	11.5 ± 3.6	11.2 ± 3.5	0.374
** Aesthetics**	15.6 ± 2.3	17.6 ± 2.8	20.1 ± 2.3	18.8 ± 3.2	21.9 ± 3.2	27.7 ± 2.9	<0.001
**Emotional state**	9.2 ± 2.0	11.7 ± 2.6	15.0 ± 2.8	10.8 ± 2.0	12.7 ± 2.1	19.3 ± 2.0	<0.001
** Total score**	55.1 ± 6.5	56.8 ± 7.1	55.9 ± 6.6	59.0 ± 5.3	62.6 ± 5.8	61.8 ± 5.5	0.005

**Data are mean ± SD,** **ESWT:** Extracorporeal Shock Wave Therapy, ** CCVUQ:** Charing Cross Venous Ulcer Questionnaire a Represents P-values obtained from the time×group interaction analysis.
